# Effect of Test Portion Mass on Vitamin A Testing in Animal Feed Materials

**DOI:** 10.1093/jaoacint/qsab158

**Published:** 2021-12-11

**Authors:** H Dorota Inerowicz, Lawrence Novotny, Charles A Ramsey, Ken L Riter, Michele Swarbrick, Nancy Thiex

**Affiliations:** Purdue University, Office of the Indiana State Chemist, 175 South University St, West Lafayette, IN, USA; (Retired), South Dakota State University, 928 8th St, Brookings, SD, USA; EnviroStat, Inc., PO Box 339, Vail, AZ, USA; Nestle Purina PetCare Co., NP Analytical Laboratories, One Checkerboard Sq, St. Louis, MO, USA; Minnesota Department of Agriculture, Laboratory Services, 601 Robert St North, St. Paul, MN, USA; Thiex Laboratory Solutions, 46747 214th St, Brookings, SD, USA

## Abstract

**Background:**

Vitamin A test results have historically been notorious for poor repeatability and reproducibility. This problem has been discussed at length in Association of American Feed Control Officials Laboratory Methods and Services Committee meetings.

**Objective:**

The objective of this work was to assess the effect of test portion mass on the repeatability of vitamin A test results.

**Methods:**

The study was conducted in two parts. In Part I, fundamental sampling error (FSE) was determined experimentally through replicated (*n *=* *16) vitamin A testing of three animal feed materials. The testing followed rigorous test portion selection for 10 g and 100 g test portions. In Part II, FSE calculations were made (*1*) using theoretical equations based on vitamin A as a liberated analyte and (*2*) on representing the particles in feed materials. Particle size characterization of vitamin A ingredients was estimated by microscopy and further evaluated by particle size analysis.

**Results:**

RSDs, % for vitamin A determinations ranged from 10.5–24.7, and 2.26–10.7 for 10 g and 100 g test portions, respectively. FSE calculated for Ingredient A ranged from 18.3–101% and 5.79–32.0% for 10 g and 100 g test portions, respectively, and for Ingredient B, ranged from 10.2–56.2% and 3.21–17.8% for 10 g and 100 g test portions, respectively.

**Conclusion:**

Test portion mass has a substantial impact on FSE and is an important factor in controlling the random error in vitamin A testing. FSE equations are useful to approximate minimum test portion mass.

**Highlights:**

Vitamin A method development should use theoretical predictions and experimental verification to guide test portion mass. Strategies to deal with the larger test portion masses will be key to validating new methods.

##  

Vitamin A is a fat-soluble vitamin and an essential nutrient required for the growth and maintenance of all vertebrates. Synthetic all-*trans* retinyl acetate is used to supplement most animal feeds. Beta-carotene may also be used as a source of vitamin A, but its activity varies widely across animal species with almost no activity in cats (1). To survive extrusion and other feed manufacturing processes and on-farm storage conditions, retinyl acetate must be encapsulated to protect vitamin A from oxygen, light, and heat (2, 3). During ingredient production, retinyl acetate is dissolved in oil with an antioxidant, then encapsulated in a gelatin beadlet, which may be cross-linked, with a starch coating (4). Encapsulated vitamin A must remain bioavailable to the target animal, which should be confirmed through feeding studies.

From a regulatory perspective, vitamin A is often the only vitamin with a guaranteed analysis on the feed label. As such, testing for vitamin A is of particular interest to the feed regulatory community. Currently, there is only one AOAC *Official Method*^SM^, Method **974.29,** for vitamin A in animal feed and pet food (5). This colorimetric method has a number of limitations: it utilizes hazardous reagents (antimony trichloride with chloroform or trifluoroacetic acid with methylene chloride); is susceptible to interferences; is time-consuming and laborious; and requires great operator skill. AOAC *Official Methods*^SM^ for vitamin A in food and infant formula (6, 7) are not applicable to animal feed and pet food without significant modification, due to encapsulation of the vitamin, the unique ingredients used, and significant interference from the complex feed matrix.

There are several “unofficial” vitamin A methods either published (8–11) or unpublished in-house methods (12, 13) for testing animal feed and pet food. Extractions include either room temperature or hot saponification with various antioxidants and supercritical fluid extraction. Extracts are cleaned using liquid–liquid partitioning and solid-phase extraction. The measurement of vitamin A includes a wide array of analytical techniques: HPLC and UHPLC with UV and fluorescence detection, LC–MS, and supercritical fluid chromatography–MS.

As a result of the frequent use of HPLC in-house methods in regulatory and industry laboratories, the Association of American Feed Control Officials (AAFCO) Laboratory Methods and Services Committee formed a Fat-Soluble Vitamins Working Group to examine the possibility of establishing one of the HPLC-based vitamin A methods currently in use as an official method. Methods were solicited from working group participants for a round-robin study to evaluate the performance of the methods. Surprisingly, from studies conducted in 2015 and 2017, at least half of the study materials showed that the repeatability of the method was greater than reproducibility with each laboratory using different analytical methods. Extra care taken to minimize distributional heterogeneity of the study materials in the second study did not change the outcome.

Sources of error in vitamin A analysis has been a topic of interest for over half a century (14–16) with test portion weight as one of these concerns. Since vitamin A is encapsulated, it has a relatively large particle size, compared to other analytes, that is not reduced during comminution. It is added to feed as a liberated analyte, introducing large compositional heterogeneity, which makes selection of the test portion more difficult and test portion mass critical. At lower concentrations, the spatial distribution of vitamin A in the feed material becomes an increasingly challenging problem to control. Error in the selection of the test portion is reflected in the test results, which is often misattributed to the analytical method rather than the test portion selection. It should be noted that for AOAC *Official Method* **974.29** specially prepared samples were fortified with crystalline retinyl acetate for the multi-laboratory validation (17), so the effects of encapsulation and the discrete particle size of vitamin A were not observed in the study results. This was a serious fault of the multi-laboratory validation.

The Theory of Sampling (TOS) was developed by Pierre Gy from the 1950s to the early 2000s (18, 19) and is a universal sampling theory that describes the sample mass, number of increments and sample correctness to achieve a representative sample. The principles of the theory describe two types of random error: fundamental sampling error (FSE) as a function of compositional heterogeneity; and grouping and segregation error as a function of the distributional heterogeneity. In addition, it describes the techniques needed to control systematic error to achieve sample correctness. The practical application of the TOS to food and agricultural materials has been described in two recent documents, *GOODSamples* (20) and *GOOD Test Portions* (21).

The goal of this study was to determine if test portion mass is a significant contributor to the large error observed in previous vitamin A studies. Experiments were performed to compare repeatability in vitamin A results for 10 g test portions and 100 g test portions in three animal feed products. Experimental data were compared to TOS calculations for fundamental error.

## PART I–Repeatability for Vitamin A Determinations in Three Animal Feed Products Using 10 g and 100 g Test Portions

 

## Experimental

### Study Materials

Animal feeds containing vitamin A were used to determine the repeatability associated with test portion mass by testing vitamin A in the feeds. The three feeds were selected based on desired levels of vitamin A and varying physical characteristics typical of feed in the marketplace. They were purchased from feed distributors in Brookings, SD, and consisted of a pelleted poultry conditioner (Feed 1), a texturized feed intended for dairy and beef cattle (Feed 2), and a mineral mix intended for cattle on pasture (Feed 3). Feed 1 was guaranteed at 10 000 IU/lb (22 000 IU/kg) Vitamin A; Feed 2 at 12 500 IU/lb (27 500 IU/kg) Vitamin A; and Feed 3 at 100 000 IU/lb (220 000 IU/kg) Vitamin A.

### Selecting 10 g and 100 g Test Portions for Vitamin A Testing

**Figure 1.**
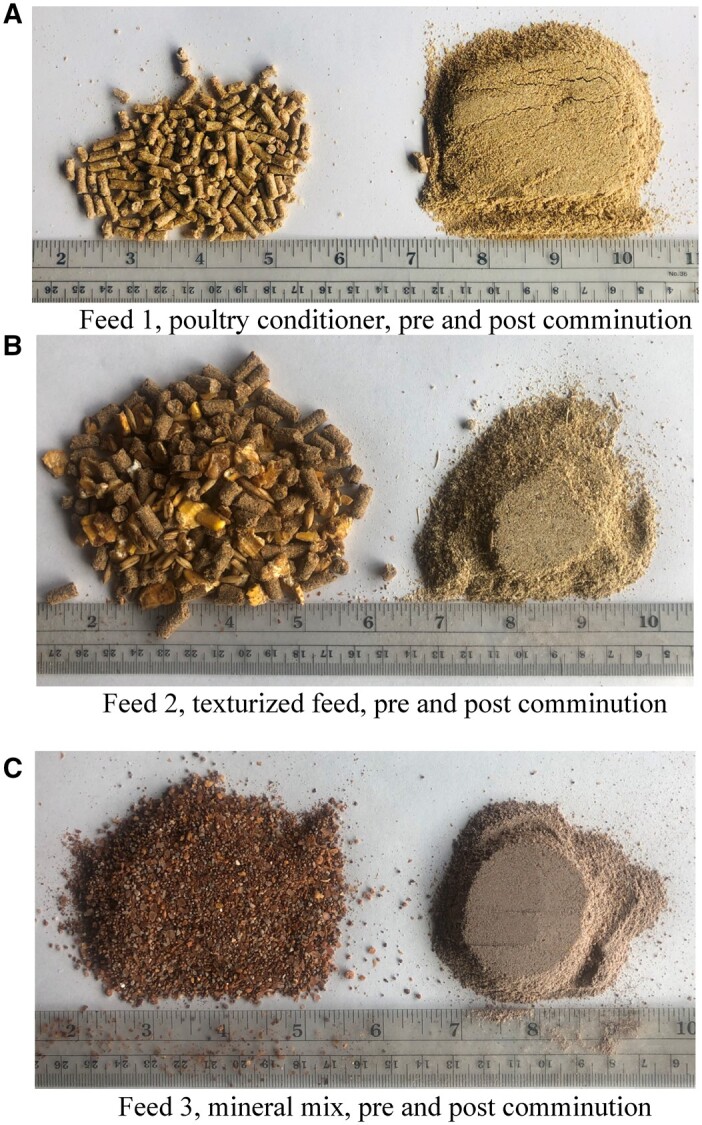
(A–C) Images of feed materials pre- and post-comminution.

**Figure 2.**
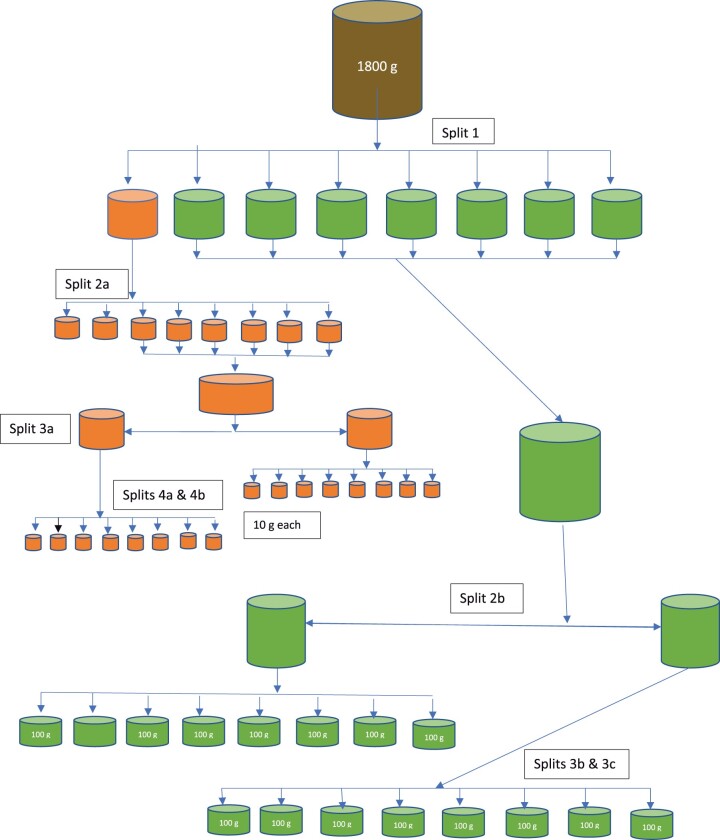
Splitting design to obtain test portions for vitamin A testing.

For all three animal feed materials, approximately 1800 g was removed from the original packaging for use in the study and the weight recorded. Feed 2 was placed in a –20°C freezer overnight to facilitate comminution. All were comminuted using a Retsch ZM200 centrifugal mill (Newtown, PA) using a 12-tooth stainless grinding rotor and a 1 mm ring screen (www.Retsch.com), with Feed 2 being removed from the freezer immediately before comminution. Each of the materials was ground in approximately 100 g portions, stopping the mill each time to empty the collection pan into a large bottle and allowing the mill to cool. Final weight after comminution was recorded. Images of the three feed materials pre- and post-comminution are provided in Figure  1A–C. The comminuted material was mixed using a Paul Schatz motion (rotating figure-of-eight motion). Test portions of approximately 10 g and 100 g (Table  1) were obtained using the sampling design outlined in Figure 2. Mixed material was transferred in portions to the hopper of a Fritsch Laborette 24 vibratory sample feeder (Fritsch Milling and Sizing, Inc., Pittsboro, NC), and slowly fed into a Fritsch Laborette 27 rotary splitter equipped with 8 × 500 mL bottles (Split 1). The weights of the eight splits were recorded for each material (*See*  Table  1).

**Table 1.** Masses for splits generated in selection of test portions for vitamin A testing

Feed material	Initial mass, g	Mass after comminution, g	Mass of initial eight splits, g	Mass of final ∼10 g test portions, g		Mass of final ∼100 g test portions, g	
Feed 1 poultry conditioner	1800.2	1793.5 (6.7 g lost)	231.4^b^	10.22	10.14	89.88	93.96
			223.0^a^	9.12	10.53	98.32	97.14
			225.2^b^	10.25	10.16	94.59	90.70
			220.6^b^	10.63	10.12	99.11	97.77
			227.1^b^	10.04	10.28	96.79	93.70
			220.0^b^	10.25	10.02	98.57	97.66
			221.1^b^	10.26	10.16	94.70	99.67
			217.2^b^	9.65	10.55	99.31	97.69
Feed 2 texturized feed	1801.0	1787.3 (13.7 g lost)	220.0^d^	10.89	10.49	94.84	97.66
			225.1^c^	10.11	10.03	97.27	96.95
			224.0^d^	10.73	11.09	96.16	96.23
			220.8^d^	9.96	10.34	97.87	97.45
			224.0^d^	10.66	10.46	96.55	97.50
			222.4^d^	10.99	10.91	95.39	95.60
			224.1^d^	10.41	10.50	97.39	98.22
			219.0^d^	10.89	10.96	95.83	97.06
Feed 3 mineral mix	1800.8	1791.8 (9.0 g lost)	223.8^f^	14.35	14.01	97.61	96.89
			224.2^f^	14.03	13.94	96.72	97.44
			225.4^e^	14.26	14.18	96.53	99.31
			227.3^f^	14.14	14.17	96.49	98.11
			223.0^f^	13.81	14.20	96.05	98.18
			222.6^f^	14.29	13.83	98.87	95.37
			223.3^f^	13.83	14.52	97.54	97.62
			219.5^f^	14.34	13.74	98.42	95.99

a Selected to yield 10 g test portions. Weights of four combined alternating subsequent splits were 81.4 and 81.4 g.

b Combined to yield 100 g test portions. Weight after combining, 1575.0 g. Weights of four combined alternating subsequent splits were 755.1 and 787.6 g.

c Selected to yield 10 g test portions. Weights of four combined alternating subsequent splits were 82.2 and 84.7 g.

d Combined to yield 100 g test portions. Weight after combining, 1555.0 g. Weights of four combined alternating subsequent splits were 755.7 and 776.3 g.

e Selected to yield 10 g test portions. Weights of four combined alternating subsequent splits were 113.1 and 113.4 g.

f Combined to yield 100 g test portions. Weight after combining, 1560.7 g. Weights of four combined alternating subsequent splits were 778.5 and 781.2 g.

- *Obtaining the ∼10 g test portions.—*One of the eight initial split bottles was split again into 8 × 28 g portions using the Fritsch Laborette 27 rotary splitter described above to yield eight new splits (Split 2a). Six (Feed 1 and Feed 2) or eight (for Feed 3) of the eight bottles were combined (and weight recorded) and the combined mass was split into eight portions of approximately 20 g each (Split 3a). Four alternating bottles were combined (and the weight recorded) and the remaining four combined (and the weight recorded). Weights were taken after each process to monitor for mass recovery error. Each of the combined masses was split into eight portions using the equipment described above to obtain 16 portions of ∼10 g each (and weights recorded for all resulting 16 test portions: Spilt 4a and 4b). Each portion was placed in a zip-lock bag, labeled, and stored in a –20°C freezer (*see*  Table  1 and Figure  2).
- *Obtaining the ∼100 g test portions.—*The remaining seven initial split bottles from Split 1 above were combined (and the mass recorded) and split into eight portions of ∼200 g each (Split 2b). Four alternating portions were combined (and the weight recorded) and the remaining four portions were combined (and the weight recorded). Weights were taken after each process to monitor for mass recovery error. Each of these combined masses was split into eight portions to obtain 16 portions of ∼100 g each (and weights recorded for all resulting 16 test portions; Spilt 3b and 3c). Each portion was placed in a zip-lock bag, labeled, and stored in a –20°C freezer *(see*  Table  1 and Figure  2).

### Vitamin A Testing

Vitamin A was determined using the method as detailed in the poster presentation available on the AAFCO web site (12). Test portions were transferred into amber high-density polyethylene containers and weights recorded. An aliquot of pyrene was added to each container to serve as an internal standard. Solutions of 0.6 mg/mL pyrogallol in ethanol and 50% (w/w) potassium hydroxide were added to each container which were then placed on a reciprocating shaker overnight at a moderate speed for saponification at room temperature. Calibration standards were saponified in the same manner. Purity and concentration of the vitamin A stock solution was determined using a spectrophotometer (16). Complete details of the method are available in the reference.

The following day, the saponicate was partially neutralized with 0.35 g/mL acetic acid in acetonitrile then placed back on the reciprocating shaker for approximately 30 min to mix. An aliquot of neutralized saponicate was transferred to a centrifuge tube then centrifuged at 500 average relative centrifugal force (rcf_ave_; 2000 revolutions per minute) for 10 min to settle solids. A 3.0 mL portion of supernatant was transferred to a second centrifuge tube containing 2.0 mL acetonitrile and mixed. Additional dilutions, if needed, were made using acetonitrile. Diluted extracts were filtered and then analyzed on an Agilent 1200 HPLC with the following conditions:

- *Column*.—Kinetex EVO C18, 100 × 4.6 mm (Phenomenex, Torrance, CA).
- *UV wavelength*.—326 nm.
- *Mobile phase*.—Methanol–water (82 + 18, by volume)
- *Flow rate*.—1.0 mL/min
- *Injection*.—20 μL.

Test portions were stored at −20°C for 6 months before analysis after receipt. Test portions were analyzed in six batches: eight 10 g and eight 100 g in each batch. The 96 vitamin A determinations were completed within a 2-week time frame.

## Results

###  

The average vitamin A in Feed 1 was found to be 6052 IU/kg using 10 g test portions and 5721 IU/kg using 100 g test portions, which is approximately 26% of the label claim (Table  2). The RSD, % of the vitamin A results for the 10 g portions was 14.8, whereas the RSD, % for the 100 g portions was 7.82; a factor of 1.9 improvement in the repeatability.

**Table 2.** Vitamin A results in Feeds 1–3 for 10 g and 100 g test portions

Replicate ID	Vitamin A, IU/kg					
	Feed 1 poultry conditioner		Feed 2 texturized feed		Feed 3 mineral mix	
Test portion mass, g	10	100	10	100	10	100
1	6112	5898	20 875	24 476	164 762	176 587
2	5230	5748	18 851	18 443	163 719	171 192
3	5352	5654	24 575	21 794	184 971	173 370
4	4875	5779	15 140	22 853	176 246	177 037
5	6736	6223	23 810	19 640	180 409	168 987
6	7801	6346	22 685	19 642	203 235	180 078
7	6575	6430	34 162	16 716	138 778	173 772
8	7294	5923	26 550	19 186	184 375	180 673
9	6818	4926	22 687	23 999	170 451	170 817
10	5768	5490	18 112	19 503	151 056	171 569
11	5682	5153	25 915	21 724	185 745	180 100
12	4646	5283	31 337	18 976	168 742	181 044
13	6904	6181	22 925	19 556	190 880	170 998
14	5907	5228	28 815	18 182	205 806	177 503
15	5746	5870	16 339	20 145	183 401	177 366
16	5389	5400	14 127	22 279	204 112	175 273
Average^a^	6052	5721	22 930	20 450	178 500	175 400
SD^a^	893	448	5673	2181	18 670	3972
RSD, %	14.8	7.82	24.7	10.7	10.5	2.26

a Rounded to 4 significant figures.

The average vitamin A in Feed 2 was found to be 22 930 IU/kg using 10 g test portions and 20 450 IU/kg using 100 g test portions, which is approximately 75% of the label claim (Table  2). The RSD, % of the vitamin A results for the 10 g portions was 24.7, whereas the RSD, % for the 100 g portions was 10.7; a factor of 2.3 improvement in the repeatability.

The average vitamin A in Feed 3 was found to be 178 500 IU/kg using 10 g test portions and 175 400 IU/kg using 100 g test portions, which is approximately 80% of the label claim (Table  2). The RSD, % of the vitamin A results for the 10 g portions was 10.5, whereas the RSD, % for the 100 g portions was 2.26; a factor of 4.5 improvement in the repeatability.

## Discussion

Repeatability (as RSD, %,) ranged from 2.26–10.7 for 100 g test portions and from 10.5– 24.7 for 10 g test portions. Repeatability of vitamin A results were improved for all feed study materials by a factor of 1.9 to 4.5, when test portion mass was increased from 10 g to 100 g. Differences in the observed repeatability can be largely attributed to mass of the test portion since other variables were carefully controlled in the study.

Average test results for Feed 2 and Feed 3 approximated the product’s guaranteed vitamin A activity; however, the results were approximately 20% the guaranteed vitamin A activity for Feed 1. Possible scenarios (underformulation, degradation of the vitamin A due to age or storage conditions of the feed, or loss of analyte integrity) were not investigated since it was not the goal of the study. While unexpected, the low values for vitamin A in Feed 1 have no impact on the results or conclusions of this study.

For these experiments test portions were selected using best practices (20, 21), such as comminution of the entire laboratory sample, use of a rotary splitter for mass reduction and Paul Schatz motion for mixing. Testing laboratories that do not use these best practices will experience a greater magnitude in vitamin A repeatability than reported here.

Experimental results will be discussed in more detail when comparing to theoretical calculations in Part II.

## PART II: Calculation of Fundamental Sampling Error for Selection of 1, 5, 10, 50, 100, and 200 g Test Portions

 

## Experimental

In addition to empirical testing, the TOS can be used to estimate the minimum test portion mass required to meet sample quality criteria (SQC). In this experiment, the factors to calculate the FSE for various test portion masses are estimated. There are two FSE calculations that are examined: (1) FSE based on representing the liberated vitamin A ingredient and (2) FSE based on representing the various particle sizes in the comminuted feed material. Finally, the theoretical results are compared to the experimental results (rounded) from Part I.

### Study Materials

Animal feeds from Part I were also used in Part II. Additionally, vitamin A ingredients were obtained to make FSE estimates from the material properties of the ingredients. Encapsulated vitamin A ingredients suitable for feed manufacturing were obtained from two independent vitamin manufacturers. Both ingredients contained approximately 1 000 000 IU/g of vitamin A as retinyl acetate. Neither vitamin manufacturer was involved in this study.

### Characterization of the Animal Feed for Density

The animal feeds were characterized for density. A portion of each study material was passed through a Fritsch Laborette 27 rotary splitter having 8 × 500 mL bottles to obtain a split of mass between 100 and 150 g. This split was comminuted through a Retsch ZM200 centrifugal mill using a 12-tooth stainless grinding rotor and a 1 mm ring screen, and the resulting material was mixed in a Glen Mills Turbula mixer (Clifton, NJ), which employs the Paul Schatz motion, for >1 min. Density was determined by transferring portions of the final comminuted material with a scoopula into a dry, tared 100 mL graduated cylinder. The material was allowed to settle and layer in the graduate without tapping or compacting techniques, and visually examined for the absence of air pockets. The volume (cm^3^) and mass (g) of the material were recorded and density calculated from an average of three measurements. Density (*n *=* *3) results are: Feed 1, 0.4927 ± 0.0110 g/cm^3^; Feed 2, 0.4600 ± 0.0049 g/cm^3^; and Feed 3, 0.9506 ± 0.0154 g/cm^3^.

### Characterization of Particle Shape, Particle Size Range, and Density of Vitamin A Ingredients

The vitamin ingredients were characterized for particle shape, particle size range, and density. The particles were examined under a microscope for size and size range, using a Leica M205C Microscope (Buffalo Grove, IL) set at 0.78 magnification. The density of each commercial vitamin ingredient was determined by transferring a portion of the material to a dry, tared 25 mL graduated cylinder. The volume and mass of the material was recorded. Density measurements were performed in triplicate.

For each of the two commercial vitamin ingredients, a visual examination for particle size range was conducted as follows. Following mixing using the Paul Schatz motion, a small portion of a few grams was removed onto white butcher paper and shaken back and forth 10 times on the paper. It was visually evident that different size particles were present, including fines, and that more testing was needed.

**Figure 3.**
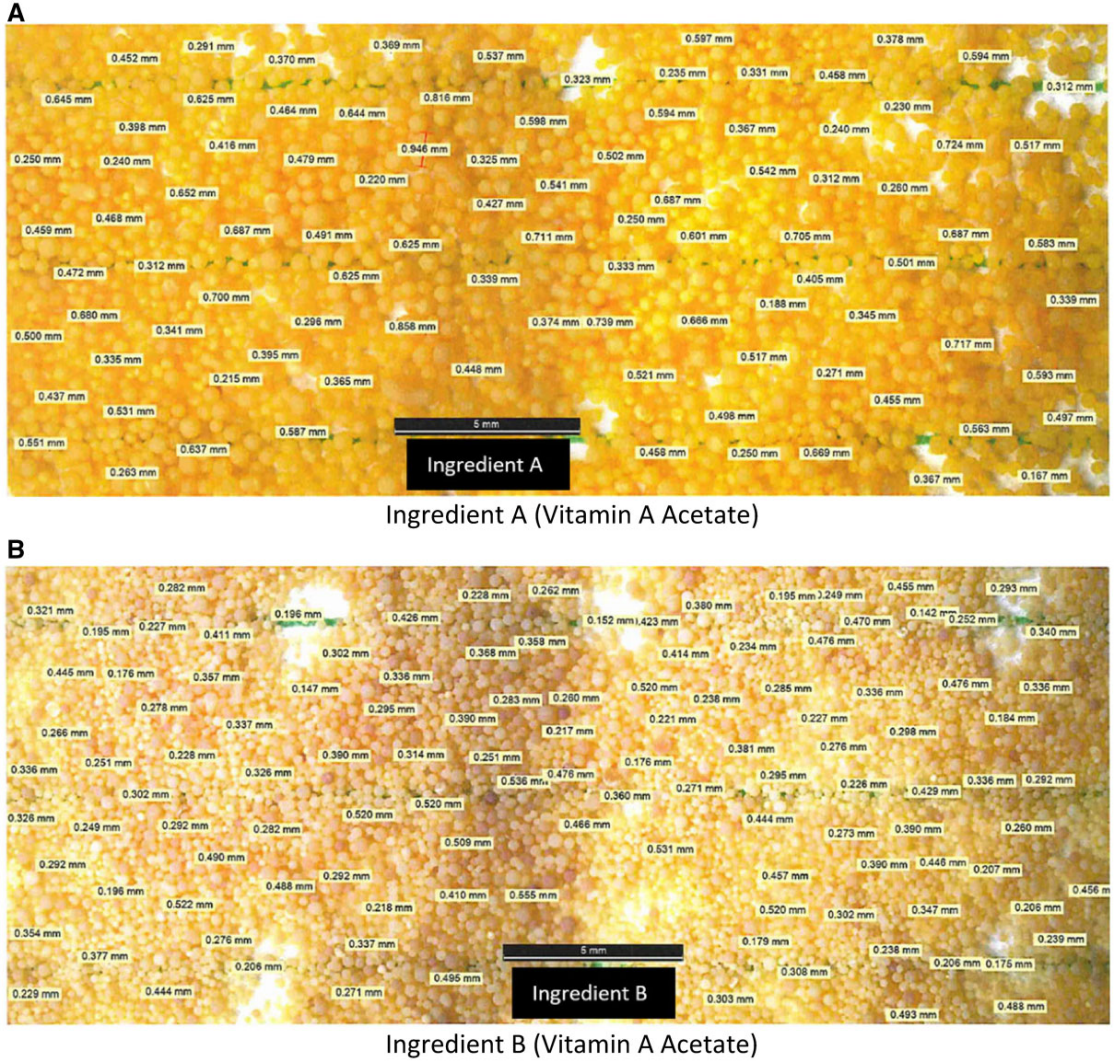
(A–B) Microscopic examination of vitamin A acetate ingredients.

A portion of each commercial vitamin A ingredient was selected for further examination after mixing the entire contents using a Paul Schatz motion, pouring on butcher paper to form a one-dimensional slab cake, and then 20 cross section aliquots (0.3–0.5 g) were removed and containerized into individual amber glass vials. Each vial was viewed under a microscope where one dimension for ∼100 particles was measured and recorded. The data is summarized in Table  3 and microscopy images are provided in Figure  3A and B. Based on the range of particle sizes observed, it was decided that detailed comprehensive particle size analysis was needed.

**Table 3.** Results of microscopic particle size measurements by microscopic examination

Vitamin ingredient	Initial mass, g	Density, g/cm^3^ @ 21.9°C^c^	Number of particles measured	Particle size measurements ^a,b^				
				Average, mm	Median, mm	Minimum, mm	Maximum, mm	SD
A	221.0	0.60	2074^a^	0.466	0.456	0.065	1.179	0.156
B	109.5	0.63	2415^b^	0.333	0.323	0.047	0.738	0.102

a Particle size measurement by microscopic examination. *n* = 20. Test portion masses ranged approximately from 0.3 to 0.5 g for Ingredient A.

b Particle size measurement by microscopic examination. *n* = 20. Test portion masses ranged approximately from 0.3 to 0.5 g for Ingredient B.

c *n* = 3.

### Particle Size Analysis

Vitamin A acetate ingredients were submitted to Microtrac Particle Size Analysis Laboratory (York, PA). Particle size analysis was performed using newer technology, dynamic image analysis, in addition to the traditional laser diffraction method on the Microtrac SYNC 3R. The dynamic image analysis results were used in establishing FSE variables. Select pages from the particle size analysis report are found in online Supplemental Appendix A, with values utilized in purple boxes. The imaging data for D95 (*see*  Supplemental Appendix A) was used to estimate the *d_0.95 _*variable for Equation 1. Imaging data was available based on volume and on number of particles. Data based on volume was used since volume is more strongly correlated with mass. The width/length (W/L) at D95, also from imaging analysis, was used to estimate the shape factor,* f.* The W/L changes with particle size, and since the shape at D95 is the most influential, it was used to estimate the shape factor, *f*. Table  4 summarizes the data from the report used to estimate variables for the FSE calculations.

**Table 4.** Data from reported particle size analysis used to estimate variable for FSE calculations

Ingredient	D95, µm	W/L^b^ or* f*
Ingredient A	684.62^a^	0.7
Ingredient B	469.46^a^	0.7

a D95 rounded to 2 significant figures before use in FSE calculations.

b Interpolated between values encompassing D95 and rounded to 1 significant figure.

### Calculations of Fundamental Sampling Error Based on Representing the Liberated Vitamin A Ingredient

Equations that describe relationships in the TOS include a number of variables. One of these variables is the liberation factor, *l*, which describes the degree of liberation of the analyte from the matrix in which it is contained. The maximum degree of heterogeneity is achieved when the analyte of interest is completely liberated (*l *=* *1). As naturally occurring vitamin A (retinol) is most likely destroyed or removed during rendering and feed manufacturing, the encapsulated vitamin A ingredient is assumed to be the only significant source of vitamin A in the finished feed or dry pet food. Thus, for the purposes of FSE calculations described later, the case for *l *=* *1 will be assumed.

FSE estimations were calculated for two commercial vitamin A acetate ingredients from different vitamin manufacturers known to be added to animal feed: Ingredient A and Ingredient B. The calculations were made for final feed concentrations of 5700, 20 400, and 175 000 IU/kg and for multiple test portion masses for each ingredient.

The equation for estimation of the variance of FSE to represent a liberated analyte, such as vitamin A, is:

(1) $$s_{\mathrm{FSE}}^{2}=\;\frac{\mathrm{cfg}d^{3}}{m_{s}}$$

where *c* = mineralogical factor, expressed in g/cm^3^; *f* = particle shape factor, dimensionless, describing the deviation from the ideal shape of a cube; *g* = granulometric factor, dimensionless, describing the range of particle sizes in the material; *d* = largest particle diameter, expressed in cm, defined as the square mesh screen that retains 5% of the mass of the material and expressed as *d_0.95_* below; and *m_s_* = mass, expressed in g, of the selection portion (18).

The mineralogical factor, c, is estimated for the liberated vitamin as:

(2) $$c\approx \frac{\lambda_{m}}{a_{L}}$$

where *a_L_* = proportion of the liberated material to the entire mass expressed as a fraction of one; and λ_*m*_ = density of the liberated material, expressed in g/cm^3 ^(18). Densities were determined for the vitamin A ingredients as follows: λ_*m*_ = 0.63 g/cm^2^ for Ingredient A and 0.60 g/cm^2^ Ingredient B. *a_L_* was calculated for Ingredient A and Ingredient B, each incorporated into animal feed at 5720, 20 400, and 175 000 IU/kg (which are the potencies obtained from the average 100 g test portion results for Feed 1, 2, and 3 from Part I of this study). Unit conversions make the *a_L_* calculations confusing; therefore, conversions and results are provided in Table  5.

*d* *=* *d_0.95_* was obtained from the particle size analysis report and is the sieve size that retains 5% of the mass of the material. *d *=* *0.068 cm for Ingredient A; d = 0.047 cm for Ingredient B (rounded to two significant digits).

*d_0.05_* was obtained from the particle size analysis report and is the sieve size that passes 5% of the mass of the material. *d_0.05_ =* 0.028 cm for Ingredient A; 0.017 cm for Ingredient B.

*f =* estimated as 0.7 for both Ingredient A and Ingredient B. *f* is the volume correction factor for converting the volume of a cube that passes the square mesh sieve to the actual volume of the particle passing the square mesh sieve. For instance, a 2 mm cube will pass a 2 mm sieve and have a shape factor of 1 while 2 mm diameter sphere will pass a 2 mm square sieve, but only has a volume of 0.52 relative to the 2 mm cube, thus the sphere has a shape factor of 0.52. While the vitamin A particles are close to spherical, the shape is slightly elongated with the magnitude of the elongation varying by size class. This elongation yields a shape factor larger than that for a perfect sphere. For this study, we used the width/length of the *d_0.95_* size class from the particle size analysis report to estimate the elongation. The shape factor was calculated by multiplying the shape factor of a perfect sphere times the elongation, defined as the length/width. While there was a slight difference between Ingredient A and Ingredient B, they both round to *f = 0.7*.

*g = 0.40* calculated from the particle size analysis*. g = (d_0.05_/d_0.95_).* While there was a slight difference between Ingredient A and Ingredient B, they both round to *g = 0.4.*

*m_s_ =* test portion mass. Calculations were performed for *m_s_ =* 1, 5, 10, 50, 100, and 200 g.

**Table 5.** Calculations for a_L_

	Vitamin A potency in ingredient			Final vitamin A concentration in feed			a_L_
Vitamin A ingredient	IU/g	µg/g	%	IU/kg	µg/kg	%	
Ingredient A	1 057 000	317 100	31.7	5720	1720	0.000172	0.00000542
Ingredient B	1 037 000	311 000	31.1	5720	1720	0.000172	0.00000553
Ingredient A	1 057 000	317 100	31.7	20 400	6120	0.000612	0.0000193
Ingredient B	1 037 000	311 000	31.1	20 400	6120	0.000612	0.0000197
Ingredient A	1 057 000	317 100	31.7	175 000	52 500	0.00525	0.000166
Ingredient B	1 037 000	311 000	31.1	175 000	52 500	0.00525	0.000169

#### Calculation  of FSE based on representing the particles present in the comminuted animal feed materials

FSE calculations were made for three animal feed study materials comminuted to pass a 1 mm (0.1 cm) screen. The simplified equation (assuming the test portion mass is less than 10% of the analytical sample mass) to represent the largest particles (largest size class) is as follows:

(3) $$s_{\mathrm{FSE}}^{2}=\frac{f\lambda }{m_{s}}\left[\frac{1}{a_{LC}}-2\right]d^{3}$$

where *f* = particle shape factor, dimensionless, describing the deviation from the ideal shape of a cube; λ = density of the feed material, expressed in g/cm^3^; *m_s_* = mass, expressed in grams, of the selection portion; *a_LC_* = 0.05 (5% largest particle sizes proportion to the entire mass); and *d *=* *0.1 cm, the largest particle diameter, expressed in cm, defined as the square mesh screen that retains largest 5% of the mass of the material (18). The test portion masses (*m_s_)* for which the FSE estimate were made in each of the three feed products were *m_s_* = 1, 5, 10, 50, 100, and 200 g.

## Results

### FSE Based on Representing the Liberated Vitamin A Ingredient

The results of the FSE calculations for liberated vitamin A from the two ingredients incorporated into the three animal feed study materials from Part I (5720 IU/kg, 20 400 IU/kg, and 175 000 IU/kg) for six test portion masses (*m_s =_* 1, 5, 10, 50, 100, and 200 g) each are provided in Table  6.

**Table 6.** The results of the FSE calculations for liberated vitamin A acetate in the animal feed study materials for six test portion masses

		Ingredient A 1 057 000 IU/g vitamin A *d = *680 µm*, f = *0.7, *g = *0.4		Ingredient B 1 037 000 IU/g vitamin A *d = *470 µm*, f = *0.7, *g = *0.4	
Concn of vitamin A acetate in feed^a^	Test portion mass (m_s_), g	$s_{\text{FSE}}^{2}$	FSE (%)	$s_{\text{FSE}}^{2}$	FSE (%)
Feed 1 poultry conditioner 5720 IU/kg (0.000172%)	200	0.0511	22.6	0.0158	12.6
	100	0.1022	32.0	0.0315	17.8
	50	0.2044	45.2	0.0631	25.1
	10	1.022	101	0.3154	56.2
	5	2.045	143	0.6308	79.4
	1	10.22	320	3.1538	178
Feed 2 texturized feed 20 400 IU/kg (0.000612%)	200	0.0144	12.0	0.00443	6.66
	100	0.0287	16.9	0.00886	9.41
	50	0.0574	24.0	0.0177	13.3
	10	0.2874	53.6	0.0887	29.8
	5	0.5748	75.8	0.1773	42.1
	1	2.874	170	0.8866	94.2
Feed 3 mineral mix 175 000 IU/kg (0.00525%)	200	0.0017	4.09	0.00052	2.27
	100	0.0033	5.79	0.00103	3.21
	50	0.0067	8.18	0.00207	4.55
	10	0.0335	18.3	0.01033	10.2
	5	0.0670	25.9	0.02066	14.4
	1	0.3349	57.9	0.1033	32.1

a Concentration as determined in Part I of this study using 100 g test portions (rounded values).

### FSE Calculations Representing the Particles Present in the Comminuted Animal Feed Materials

The results of the FSE calculations for representing the particles present in the three animal feed study materials comminuted to pass a 1 mm sieve for six test portion masses (*m_s_* = 1, 5, 10, 50, 100, and 200 g) are presented in Table  7.

**Table 7.** FSE calculations for representing the particles present in three animal feed materials comminuted to pass a 1 mm sieve

	Feed 1 poultry conditioner (λ = 0.4927 g/cm^3^)		Feed 2 texturized feed (λ = 0.4600 g/cm^3^)		Feed 3 mineral mix (λ = 0.9506 g/cm^3^)	
Test portion mass (m_s_), g	$s_{\text{FSE}}^{2}$	FSE (%)	$s_{\text{FSE}}^{2}$	FSE (%)	$s_{\text{FSE}}^{2}$	FSE (%)
200	0.000022	0.47	0.000021	0.45	0.000043	0.65
100	0.000044	0.67	0.000041	0.64	0.000086	0.93
50	0.000089	0.94	0.000083	0.91	0.000171	1.31
10	0.000443	2.11	0.000414	2.03	0.000856	2.93
5	0.000886	2.98	0.000828	2.88	0.001711	4.14
1	0.00443	6.66	0.004140	6.43	0.008555	9.25

### Comparison of FSE Calculations

The FSE estimates resulting from the two calculations were compared to determine whether the liberated vitamin in the feed materials, or the particle sizes of the carrier feed matrix is the dominant factor affecting FSE. The larger of the two minimum masses is required as the test portion mass necessary to control the FSE for the determination of vitamin A in the feeds. In all cases, for a given test portion mass, the FSE associated with representing the particle sizes is much smaller than the FSE associated with representing the liberated vitamin A in the feed. Therefore, the minimum mass needed to represent the liberated vitamin will be the minimum mass required for the test portion and will be used in this study.

## Discussion

### Comparison of Experimental RSD, % to Calculated FSE (as %RSD) Values

Calculated FSE (as RSD, %) based on incorporation of Ingredients A and B into Feeds 1, 2, and 3 are shown with the corresponding experimental RSDs, % from Part I in Table  8. Excellent agreement between calculated FSE and experimental RSDs for Ingredient B and Feed 2 and Feed 3 were observed. However, calculated FSE for either Ingredient A or B are in poor agreement with experimental RSD values for Feed 1. The better than anticipated RSDs lead to the authors to speculate that an ingredient of much smaller particle size than Ingredients A and B may have been used to manufacture Feed 1. A vitamin A beadlet particle size, d_0.95_, of approximately 0.02 cm would be needed to achieve the observed RSDs, which is much different than Ingredient A or B.

**Table 8.** Comparison of experimental results from Part I to theoretical results

	Feed 1 poultry conditioner, 57**2**0 IU/kg		Feed 2 texturized feed, 20 400 IU/kg		Feed 3 mineral mix, 175 000 IU/kg	
Test portion mass, g	Experimental RSD, %	Calc. FSE (RSD, %)^a^	Experimental RSD, %	Calc. FSE (RSD, %)^a^	Experimental RSD, %	Calc. FSE (RSD, %)^a^
10	14.8	101 (A)	24.7	53.6 (A)	10.5	18.3 (A)
		56.2 (B)		29.8 (B)		10.2 (B)
100	7.82	32.0 (A)	10.7	16.9 (A)	2.26	5.79 (A)
		17.8 (B)		9.4 (B)		3.21 (B)

a Calculated FSE (as RSD, %) using (Eq. 1) and using the vitamin A concentrations determined experimentally in Part I. (A) value calculated for vitamin Product A. (B) value calculated for vitamin Product B.

Error in a measurement, termed global estimation error, is comprised of primary sampling error, laboratory sampling error, and analytical uncertainty. The amount of global estimation error that can be tolerated is based upon the sampling quality criteria to assure the test result is fit for decision (20, 21). These studies do not address primary sampling error. For vitamin A analysis, this study demonstrates that TOS equations for FSE for liberated analytes can be useful for predicting RSD, %. Inputs are based on vitamin A ingredient beadlet characterization. The same equations can be used (by solving for mass) to estimate the minimum test portion mass to achieve a specified FSE. Assuming best practices in laboratory sampling procedures, the test portion mass needed to achieve a RSD, % of 10 in test results for the vitamin A concentrations observed in Feeds 1, 2, and 3 for Ingredients A–B are shown in Table  9. These test portion masses are larger than currently in practice and may require higher dilutions. However, dilution errors are insignificant compared to sampling errors and can be controlled with the use of an internal standard.

**Table 9.** Test portion mass to achieve 10% RSD among replicated tests

Vitamin A concentration			Test portion mass to achieve 10% RSD, g	
IU/kg	IU/lb	%	Vitamin A Ingredient A	Vitamin A Ingredient B
5000	2300	0.00015	1170	360
10 000	4500	0.00030	590	180
50 000	23 000	0.0015	120	36
100 000	45 000	0.0030	59	18
500 000	230 000	0.015	12	3.6
1 000 000	450 000	0.030	5.9	1.8
5 000 000	2 300 000	0.15	1.2	0.36
10 000 000	4 500 000	0.30	0.59	0.18

### Practical Considerations for Vitamin A Testing

Both the experimental results and FSE calculations show that test portion mass is critical for controlling random error (repeatability) in vitamin A testing. Repeatability is related to the physical characteristics of the vitamin A ingredient beadlets used in the manufacture of the feed and is inversely related to concentration of vitamin A in the feed. The data presented in this study are based on the use of best practices (20, 21) in obtaining the test portion. Testing laboratories that do not use these best practices will experience greater error in their vitamin A results, which underscores that laboratory personnel need to be competent in the proper laboratory sampling procedures.

To accurately predict FSE, the vitamin A ingredient beadlet used to manufacture the feed and the associated particle characteristics of the ingredient need to be known. The authors know of at least six vitamin manufacturers producing encapsulated feed-grade vitamin A used in North America and were able to obtain only two. A testing laboratory will almost never know the ingredient used in a feed material received as a laboratory sample. Even if every commercially available vitamin A ingredient was well characterized by the laboratory, the laboratory would still only have a range of minimum test portion masses to achieve a given FSE. If the ingredient is unknown, the laboratory must plan for the scenario requiring the largest mass when establishing the test portion mass.

## Conclusions

Test portion mass was shown to have a significant impact on vitamin A result repeatability, and these results are consistent with TOS calculations. This study examined a small number of animal feeds and vitamin A ingredients and carefully controlled splitting procedures. The mass required to control FSE in most animal feeds received by laboratories will be a function of the SQC (fit-for-decision) and likely be larger than what is reported in this study. Future vitamin A method studies, including an AOAC multilaboratory validation, should use theoretical predictions, as in Table  9, to guide needed test portion mass. Key to progress on a validated vitamin A HPLC-based method will be strategies to deal with the larger test portion masses required for acceptable repeatability.

## Supplemental Information

Appendixes and Supplemental Information are available on the *J. AOAC Int*. website.

## CRediT Author Statement

**H.** **Dorota Inerowicz:** Conceptualization, Formal analysis, Investigation, Methodology, Project administration, Resources, Validation, Visualization, Writing—original draft, Writing—reviewing & editing. **Lawrence Novotny**: Conceptualization, Formal analysis, Investigation, Methodology, Project administration, Resources, Validation, Visualization, Writing—original draft, Writing—reviewing & editing. **Charles A. Ramsey:** Conceptualization, Formal analysis, Investigation, Methodology, Project administration, Resources, Validation, Visualization, Writing—original draft, Writing—reviewing & editing. **Ken L. Riter:** Conceptualization, Formal analysis, Investigation, Methodology, Project administration, Resources, Validation, Visualization, Writing—original draft, Writing—reviewing & editing. **Michele Swarbrick**: Conceptualization, Formal analysis, Investigation, Methodology, Project administration, Resources, Validation, Visualization, Writing—original draft, Writing—reviewing & editing. **Nancy Thiex:** Conceptualization, Formal analysis, Investigation, Methodology, Project administration, Resources, Validation, Visualization, Writing—original draft, Writing—reviewing & editing.

## Supplementary Material

qsab158_Supplementary_Data
