# Supplementary material for: Effect of Test Portion Mass on Vitamin A Testing in Animal Feed Materials
Source: J AOAC Int. 2021 Dec 11;105(1):288–98. doi: 10.1093/jaoacint/qsab158 (PMC8824796; doi:10.1093/jaoacint/qsab158)
Supplement: qsab158_Supplementary_Data [file qsab158_supplementary_data.zip › aoac-21-0280-File006.pptx]

## Slide 1
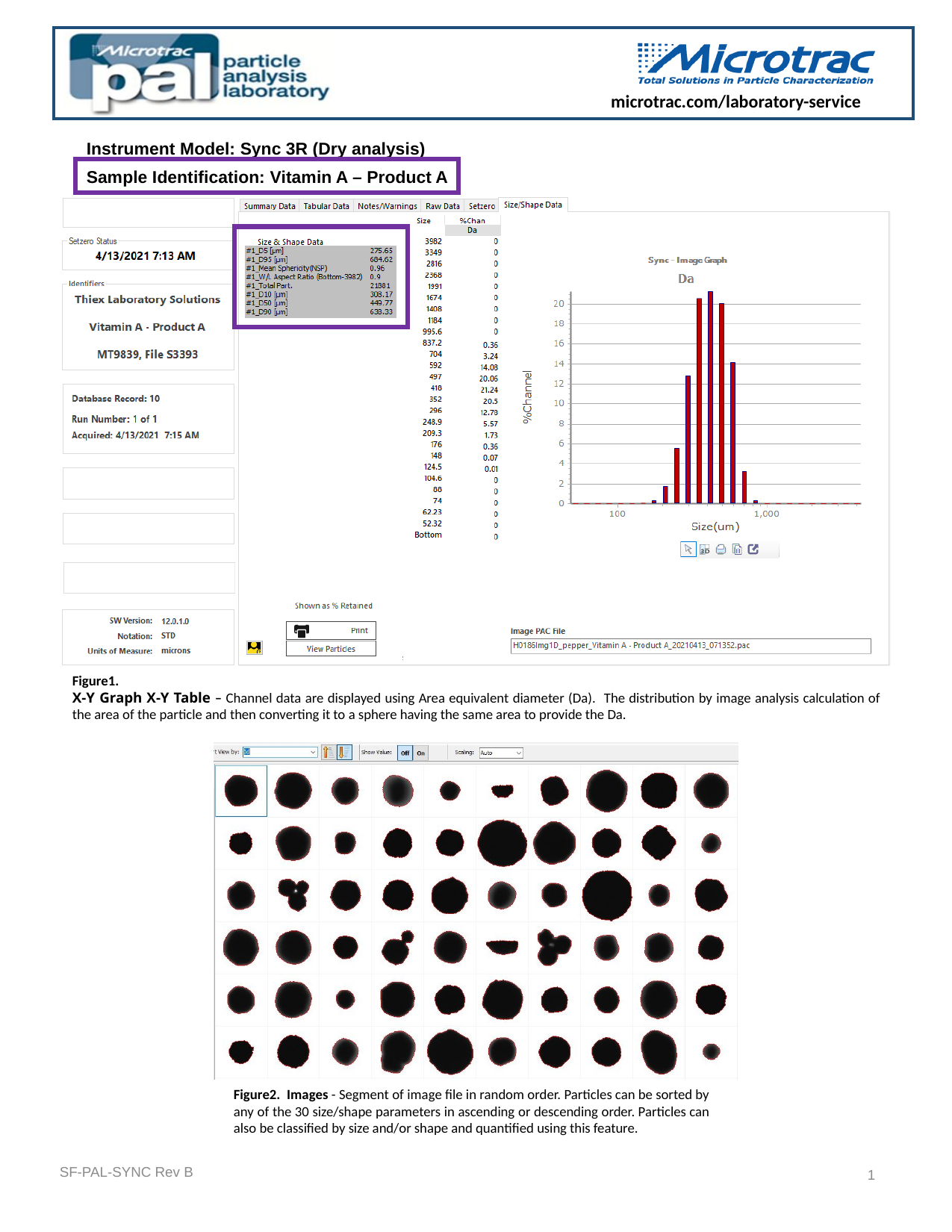

Instrument Model: Sync 3R (Dry analysis)
Sample Identification: Vitamin A – Product A
Figure1.
X-Y Graph X-Y Table – Channel data are displayed using Area equivalent diameter (Da). The distribution by image analysis calculation of the area of the particle and then converting it to a sphere having the same area to provide the Da.
Figure2. Images - Segment of image file in random order. Particles can be sorted by any of the 30 size/shape parameters in ascending or descending order. Particles can also be classified by size and/or shape and quantified using this feature.
1

## Slide 2
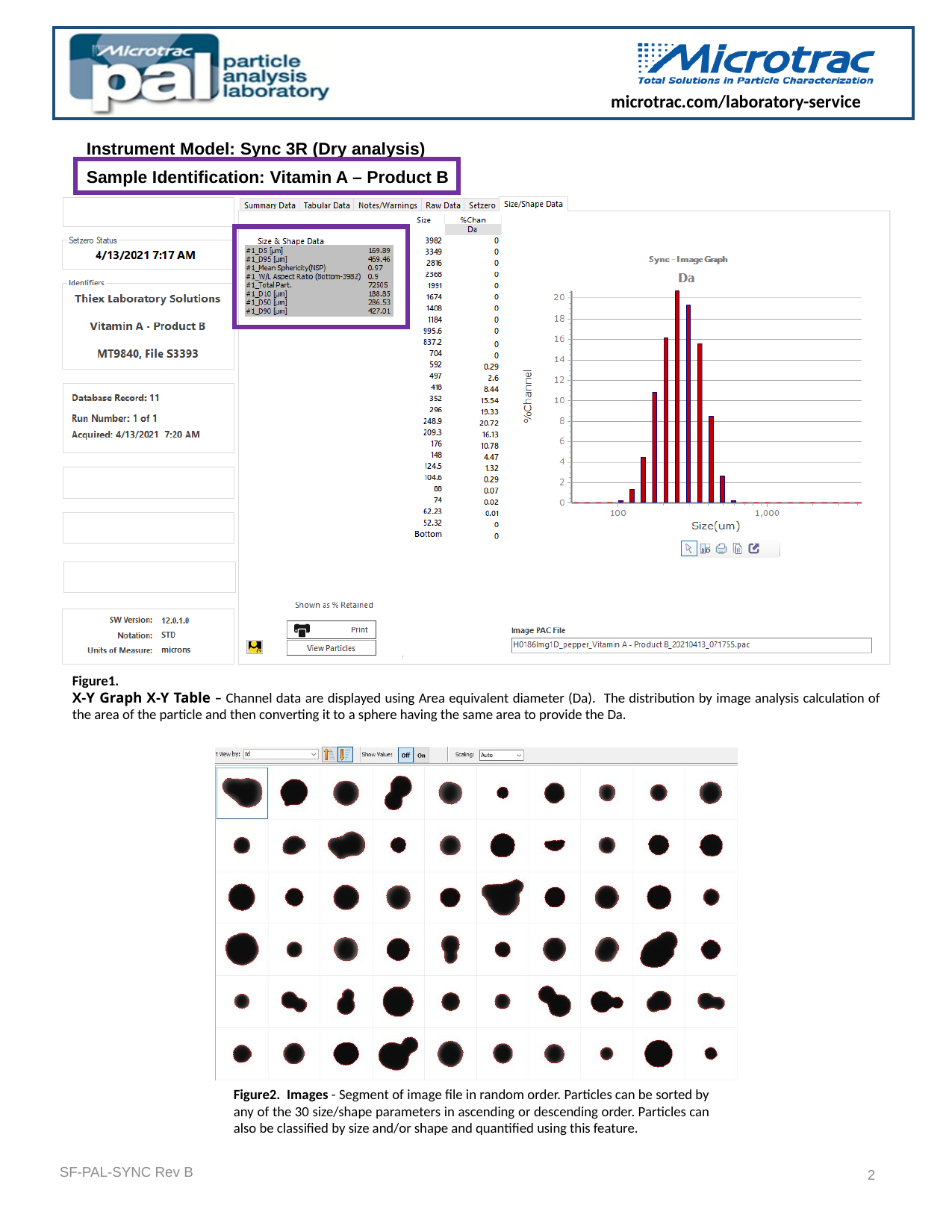

Instrument Model: Sync 3R (Dry analysis)
Sample Identification: Vitamin A – Product B
Figure1.
X-Y Graph X-Y Table – Channel data are displayed using Area equivalent diameter (Da). The distribution by image analysis calculation of the area of the particle and then converting it to a sphere having the same area to provide the Da.
Figure2. Images - Segment of image file in random order. Particles can be sorted by any of the 30 size/shape parameters in ascending or descending order. Particles can also be classified by size and/or shape and quantified using this feature.
2

## Slide 3
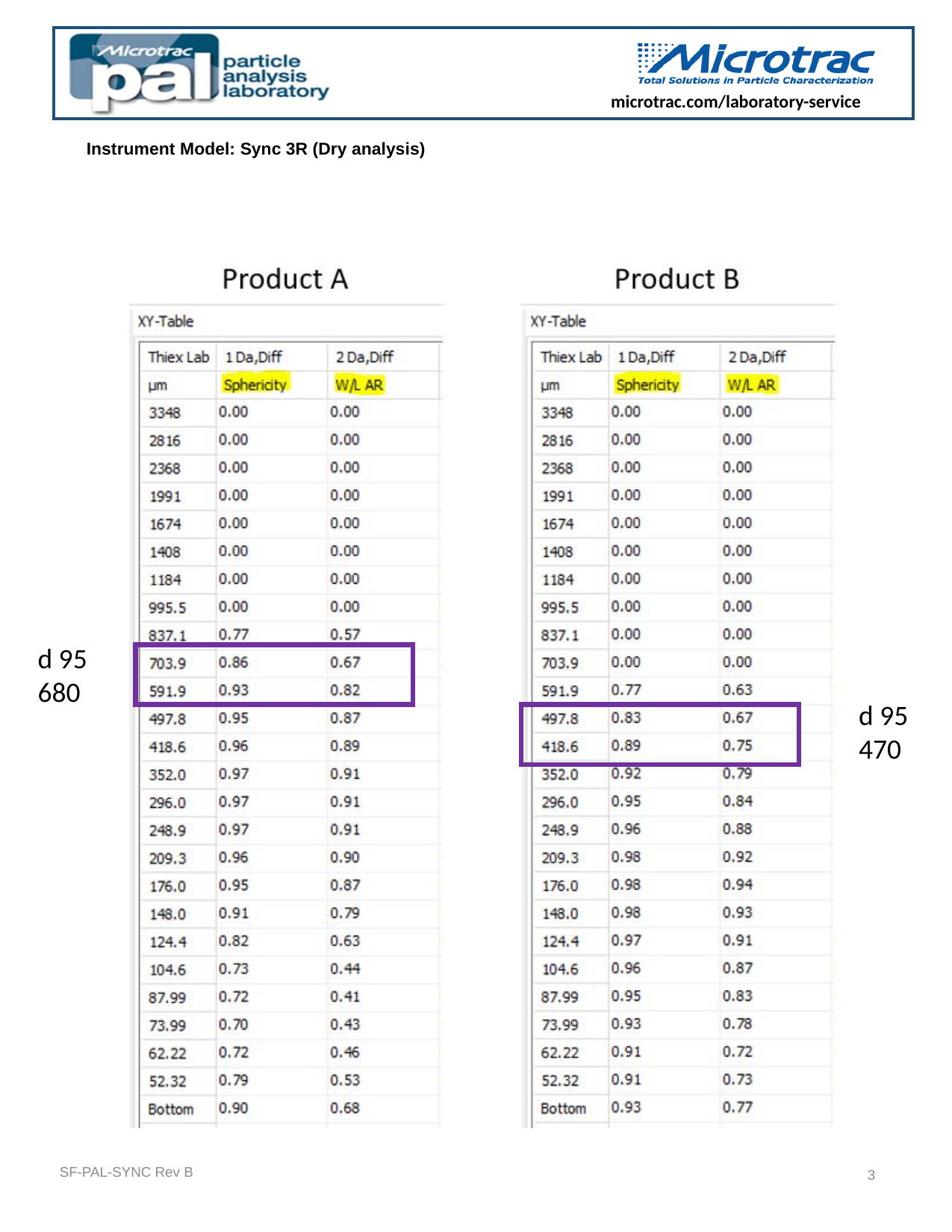

Instrument Model: Sync 3R (Dry analysis)
d 95
680
d 95
470
3
